# Association of Constipation and Geriatric Depressive Symptoms: Cross-Sectional Analysis Using Baseline Data from the JUSTICE-TOKYO Prospective Cohort Study

**DOI:** 10.3390/diagnostics15121537

**Published:** 2025-06-17

**Authors:** Hiroyuki Kiko, Daisuke Asaoka, Osamu Nomura, Yusuke Nomoto, Koji Sugano, Kei Matsuno, Yasuhiro Homma, Yuji Nishizaki, Naotake Yanagisawa, Tsutomu Takeda, Daiki Abe, Shotaro Oki, Nobuyuki Suzuki, Yoichi Akazawa, Kumiko Ueda, Hiroya Ueyama, Mariko Hojo, Akihito Nagahara, Hiroyuki Isayama, Katsumi Miyauchi

**Affiliations:** 1 Department of Gastroenterology, Juntendo Tokyo Koto Geriatric Medical Center, Tokyo 136-0075, Japan; h.kiko.yd@juntendo.ac.jp (H.K.); onomura@juntendo.ac.jp (O.N.); ynomoto@juntendo.ac.jp (Y.N.); 2 Department of Respiratory Medicine, Juntendo Tokyo Koto Geriatric Medical Center, Tokyo 136-0075, Japan; ksugano@juntendo.ac.jp (K.S.); kmatsuno@juntendo.ac.jp (K.M.); 3 Department of Orthopedic Surgery, Juntendo Tokyo Koto Geriatric Medical Center, Tokyo 136-0075, Japan; yhomma@juntendo.ac.jp; 4 Medical Technology Innovation Center, Juntendo University, Tokyo 113-8421, Japan; ynishiza@juntendo.ac.jp (Y.N.); n-yanagisawa@juntendo.ac.jp (N.Y.); 5 Department of Gastroenterology, Juntendo University School of Medicine, Tokyo 113-8421, Japan; t-takeda@juntendo.ac.jp (T.T.); d-abe@juntendo.ac.jp (D.A.); s-oki@juntendo.ac.jp (S.O.); nb-suzuki@juntendo.ac.jp (N.S.); yakazawa@juntendo.ac.jp (Y.A.); ktamaki@juntendo.ac.jp (K.U.); psyro@juntendo.ac.jp (H.U.); mhojo@juntendo.ac.jp (M.H.); nagahara@juntendo.ac.jp (A.N.); h-isayama@juntendo.ac.jp (H.I.); 6 Department of Cardiology, Juntendo Tokyo Koto Geriatric Medical Center, Tokyo 136-0075, Japan; ktmmy@juntendo.ac.jp

**Keywords:** geriatric depression scale 15, constipation, constipation scoring system, elderly, depressive symptoms

## Abstract

**Objective:** To clarify the relationship between constipation and depressive symptoms among the elderly. **Methods:** This single-center, cross-sectional study was performed using baseline data obtained at the time of enrollment in the prospective cohort of the JUSTICE-TOKYO study. Participants underwent assessments including patient profiling, drug use history, the Mini-Mental State Examination (MMSE), the Geriatric Depression Scale 15 (GDS-15), gastrointestinal-related quality of life (QOL), and the constipation scoring system (CSS). Geriatric depressive symptoms were evaluated based on GDS-15. We investigated correlations between GDS-15 scores and various abdominal symptoms and assessed risk factors for geriatric depressive symptoms using multiple regression analysis. **Results:** A total of 984 elderly participants (57% female, mean age 78.1 ± 6.1 year) were included. The GDS-15 scores were significantly correlated with body mass index (BMI) (r = −0.056) and MMSE (r = −0.092), reflex-related QOL (r = 0.253), pain-related QOL (r = 0.229), fullness-related QOL (r = 0.269), constipation-related QOL (r = 0.329), diarrhea-related QOL (r = 0.264), and CSS (r = 0.285) scores. Multiple regression analysis indicated that BMI (β = −0.069, *p* = 0.020) and MMSE (β = −0.074, *p* = 0.013), constipation-related QOL (β = 0.136, *p* = 0.002), reflex-related QOL (β = 0.126, *p* < 0.001), diarrhea-related QOL (β = 0.095, *p* = 0.006), and CSS (β = 0.098, *p* = 0.016) scores were significantly correlated with GDS-15 scores. **Conclusions:** Depressive symptoms among older individuals are associated with various abdominal symptoms, particularly constipation. However, the causality between depressive symptoms and constipation cannot be inferred due to the study’s cross-sectional design.

## 1. Introduction

In Japan, a hyper-aged society, the elderly population is projected to account for approximately 40% of the total population by 2060 [1]. Constipation becomes more common with age [2]. Constipation not only impairs quality of life (QOL) [3] but also shortens life expectancy by predisposing individuals to coronary artery disease and cerebrovascular disease [4]. Recent studies have further linked constipation to frailty [5] and sarcopenia [6], conditions commonly observed in the elderly. Chronic constipation has a complex and multifactorial etiology, including diet, stress, medications, neurological issues [7], and psychological distress [8]. There is growing evidence that changes in the intestinal microbiota contribute to constipation and constipation-related symptoms. Chronic constipation is a gastrointestinal disorder with symptoms characterized by difficulty or decreased frequency of bowel movements, hardness of stools, and/or residual stools. It can cause discomfort such as abdominal distention, abdominal pain, headache, dizziness, and loss of appetite [9]. In recent years, it has become clear that bidirectional communication between the gut and brain via the gut–brain axis has significant implications for both gastrointestinal and neurological health.

Older individuals are susceptible to developing depression [10]. Similar to constipation, depression diminishes QOL [11], reduces appetite [12], and may interfere with the management of comorbidities, potentially impacting life expectancy [13]. It also predisposes individuals to sarcopenia [14], highlighting the need for early intervention.

Central serotonin (5-hydroxytryptamine [5-HT]) plays a crucial role in the mechanism underlying the association between constipation and depression. Two distinct isozymes of tryptophan hydroxylase (Tph), Tph1 and Tph2, modulate 5-HT biosynthesis in non-neuronal and neuronal systems [15]. Although Tph2 is responsible for 5-HT production in the brain, >90% of the body’s 5-HT is synthesized in the gut via Tph1, mainly within enterochromaffin cells, which are specialized intestinal epithelial cells. This process converts 5-hydroxytryptophan (5-HTP) to 5-HT. Although the former can enter the central nervous system to produce serotonin, the latter cannot cross the blood–brain barrier under physiological conditions [16]. The gut microbiota plays a crucial role in intestinal 5-HT production by increasing Tph1 expression in colonic enterochromaffin cells [17].

However, there is limited literature addressing the relationship between constipation and depressive symptoms in older individuals. Few studies have explored this association in Japan, and even fewer have examined it in detail using tools such as the Izumo Scale or the constipation scoring system (CSS), which assess gastrointestinal-related QOL and constipation severity. We assessed the associations between constipation and depressive symptoms among the older population.

## 2. Materials and Methods

### 2.1. Study Design

This single-center, cross-sectional study was performed using baseline data obtained at the time of enrollment in the Juntendo Sarcopenia Registration of Exploring for Predictors and Prognosis in Elderly in TOKYO (JUSTICE-TOKYO) study [18], which included older outpatients (aged ≥65 years) treated at Juntendo Tokyo Koto Geriatric Medical Center between November 2020 and November 2021. The JUSTICE-TOKYO study is a prospective cohort designed to elucidate the clinical characteristics and prognosis of sarcopenia in the elderly. The study includes a 4-year follow-up, with annual evaluations (e.g., survival, falls, hospitalization, and skeletal muscle mass); the study is expected to conclude in 2025. At enrollment, baseline data (e.g., patient profiles, drug use history, abdominal questionnaires, and neuropsychological examinations) were collected. Data were prospectively recorded in the Research Electronic Data Capture (REDCap) system [19]. However, the information does not include longitudinal assessments related to constipation or depression. Therefore, in this study, we conducted a cross-sectional analysis based solely on baseline data collected at the time of enrollment in the JUSTICE-TOKYO study to examine the relationship between constipation and geriatric depressive symptoms. We investigated correlations between GDS-15 scores and various abdominal symptoms and assessed risk factors for geriatric depressive symptoms using multiple regression analysis.

### 2.2. Exclusion Criteria

Individuals fulfilling the below-mentioned criteria were excluded:

(i) Need for support during walking due to severe osteoarthritis or neuromuscular disease;
(ii) Immobility;
(iii) Delirium tremens at presentation;
(iv) Prior gastrointestinal, renal, acute cerebrovascular, coronary, hepatic, or respiratory events;
(v) Inability to complete a questionnaire interview;
(vi) Life expectancy < 1 year due to malignancy.

### 2.3. Measurement of Baseline Variables

Study Instruments: We included patients with complete data, encompassing the following:

(i) Patient Profile: Age, gender, body mass index (BMI), Brinkman index, and alcohol intake (0 = rarely drinks alcohol; 1 = drinks alcohol 1–4 days/week; 2 = drinks alcohol 5–7 days/week).
(ii) Use of Therapeutic Agents: statins, acid secretion suppressants, and laxatives.
(iii) Neuropsychological Examinations: Geriatric Depression Scale 15 (GDS-15) and Mini-Mental State Examination (MMSE) [20,21]. A shortened version of the GDS (GDS-15) was used to assess depressive symptoms. GDS-15 scores are classified as follows: scores of 0–4 are considered normal, scores of 5–9 indicate mild depression and scores above 10 indicate moderate to severe depression [22]. The MMSE is a widely used, reliable, and validated instrument used in screening for cognitive impairment. It examines a few aspects of cognition, is easily performed, and requires 5 to 10 min to administer. Its contents include orientation, attention, learning, calculation, abstraction, information, construction, and delayed recall. A high degree of correlation has been shown between this test and standard tests of cognitive function.
(iv) Gastrointestinal-Related QOL: The Izumo scale is a 15-item self-administered tool that assesses reflux, upper abdominal pain, fullness, constipation, and diarrhea on a 5-point Likert scale [23]. Domain-specific QOL impairment is scored from 0 (no impairment) to 15 (severe impairment).
(v) Constipation Severity: The CSS [24] evaluates eight items: frequency of bowel movements, painful evacuation, incomplete evacuation, abdominal pain, duration per attempt, assistance for evacuation, unsuccessful attempts at evacuation per 24 h, and duration of constipation. The score ranges from 0 to 30, with higher scores indicating more severe constipation symptoms.

### 2.4. Statistical Analysis

Quantitative data are presented as mean ± standard deviation. Non-normally distributed data (MMSE, GDS-15, gastrointestinal QOL score CSS score) are presented as the median (IQR). In terms of the clinical characteristics of the study participants by GDS-15 severity, ANOVA analysis was performed for parametric data (BMI), and Kruskal–Wallis tests were performed for nonparametric data (CSS score, reflux, pain, fullness, constipation, diarrhea, and GDS score). Correlations between the GDS-15 score and clinical parameters (age, BMI, Brinkman index, MMSE, reflux-related QOL score, upper abdominal pain-related QOL score, fullness-related QOL score, constipation-related QOL score, diarrhea-related QOL score, and CSS score) were investigated using Pearson’s correlation coefficients. For the GDS scores, residuals were visually assessed using histograms and Q–Q plots. As they appeared to be approximately normally distributed and nearly symmetrical, the application of multiple regression analysis was deemed appropriate. Multiple regression analysis was conducted to identify risk factors associated with elevated GDS-15 scores. All independent variables including patient background characteristics, cognitive function test scores, gastrointestinal-related quality of life measures, CSS scores, and medication data were initially entered into the model simultaneously, and the stepwise method was then used to refine the model. Multicollinearity was evaluated using variance inflation factors (VIFs). Statistical analyses were conducted using SPSS version 28 software (IBM Corporation). A *p*-value < 0.05 was considered indicative of statistical significance.

## 3. Results

### 3.1. Patient Characteristics

**Figure 1:**
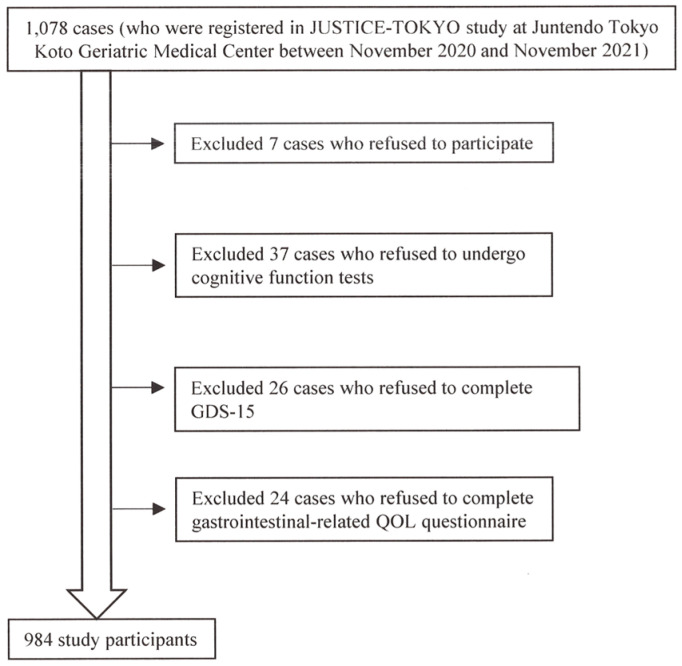
The patient selection process.

Figure 1 presents the patient selection process. Of the 1078 cases who were registered in the JUSTICE-TOKYO study at Juntendo Tokyo Koto Geriatric Medical Center between November 2020 and November 2021, 984 participants were eligible. Table 1 presents the baseline data for 984 participants (557 females [57%]; mean age, 78.1 ± 6.1 years; mean BMI, 22.9 ± 3.7 kg/m^2^; MMSE score, 27 (25, 29); GDS-15 score, 4 (2, 6); reflex-related QOL score, 1 (0, 3); upper abdominal pain score, 0 (0, 1); fullness-related QOL score, 0 (0, 3); constipation-related score, 1.5 (0, 4); diarrhea-related QOL score, 1 (0, 3); CSS score, 2 (1, 6)). Table 2 presents the clinical characteristics of the study participants by GDS-15 severity. There were differences in the various abdominal symptom scores (gastrointestinal-related QOL score, CSS score) according to the severity of GDS-15 among the three groups (*p* < 0.0001).

Of the 1078 cases who were registered in the JUSTICE-TOKYO study at Juntendo Tokyo Koto Geriatric Medical Center between November 2020 and November 2021, 984 participants were eligible.

### 3.2. Correlations Between Geriatric Depression Scale 15 Score and Various Clinical Parameters

Table 3 presents Pearson’s correlation coefficients. The GDS-15 score was significantly associated with BMI (r = −0.056) and MMSE (r = −0.092), reflux-related QOL (r = 0.253), pain-related QOL (r = 0.229), fullness-related QOL (r = 0.269), constipation-related QOL (r = 0.329), diarrhea-related QOL (r = 0.264), and CSS (r = 0.285) scores.

### 3.3. Multiple Regression Analysis for Associations Among GDS-15 Score and Other Variables

Table 4 summarizes the multiple regression analysis for associations between GDS-15 score and other variables. BMI (β = −0.069, *p* = 0.020) and MMSE (β = −0.074, *p* = 0.013), constipation-related QOL (β = 0.136, *p* = 0.002), reflex-related QOL (β = 0.126, *p* < 0.001), diarrhea-related QOL (β = 0.095, *p* = 0.006), and CSS (β = 0.098, *p* = 0.016) scores were significantly associated with the GDS-15 score.

## 4. Discussion

This is the first study from Japan to investigate the relationship between constipation and depressive symptoms in the elderly using detailed abdominal questionnaires, such as the Izumo scale and CSS. The GDS-15 score was significantly associated with BMI, MMSE score, all gastrointestinal-related QOL scores (reflux, upper abdominal pain, fullness, constipation, and diarrhea), and CSS score. Multiple regression analysis revealed that BMI and MMSE, constipation-related QOL, reflex-related QOL, diarrhea-related QOL, and CSS scores were significantly associated with the GDS-15 score.

Dykes et al. reported that, in cases of intractable constipation, three-fifths of patients showed evidence of a current affective disorder [25]. Psychological impairment was observed in 65% of patients with evacuation disorders and constipation in a tertiary care setting [26]. Constipated individuals have significantly higher anxiety and depression scores [27]. Women with constipation showed abnormal general psychosocial functioning, somatization, anxiety, depression, and feelings related to their gender role [28]. A previous study demonstrated an association between depression and the risk of chronic constipation [29]. Vu et al. identified severe depression, female sex, and sleep duration ≤ 6 h per day as significant factors associated with chronic constipation in multivariate models [30].

Central serotonin (5-hydroxytryptamine [5-HT]) plays a crucial role in the mechanism underlying the association between constipation and depression.

Vitamin B6 (pyridoxal phosphate, PLP), a coenzyme in the tryptophan-serotonin pathway, may influence depression. It is postulated that vitamin B6 deficiency can contribute to depression [31]. Hvas et al. found that low plasma PLP levels were associated with depressive symptoms [32]. The human gut microbiota can synthesize vitamins, especially B vitamins, and vitamin B6 biosynthesis is linked to certain intestinal bacteria, such as Bacteroides spp., Escherichia coli, and Bifidobacterium [33]. Studies have shown that species such as Bifidobacterium and Bacteroides may be quantitatively altered in individuals with functional constipation [34]. Ohkusa et al. found an association between gut microbiota dysbiosis and functional constipation and constipation-type irritable bowel syndrome [35]. Our previous randomized, double-blind, placebo-controlled, parallel-group superiority trial in Japan demonstrated the partial effectiveness of Bifidobacterium longum BB536 supplementation in elderly individuals with chronic constipation [36]. Short-chain fatty acids (SCFAs), including butyric acid, acetic acid, and propionic acid, are major metabolites of intestinal bacteria [37] and promote the secretion of endogenous GLP-1 from L-cells, thereby enhancing colonic peristalsis [38]. Among SCFAs, butyric acid is particularly associated with constipation [39]. Butyrate may also play a role in improving depressive symptoms by increasing Tph1 expression in intestinal epithelial cells and promoting serotonin production [40]. Sun et al. reported that sodium butyrate significantly improved depression-like behaviors in chronic, unpredictable, mildly stress-induced mice, with its antidepressant effects potentially related to the restoration of blood–brain barrier integrity and increased brain 5-HT concentrations and BDNF expression [41]. A potential mechanism linking constipation and depressive symptoms may involve gut dysbiosis and decreased SCFA production, particularly butyrate, leading to reduced 5-HTP production via Tph1. This, in turn, may reduce brain serotonin levels, contributing to depressive symptoms. Recent studies have indicated that probiotic intake may help prevent or alleviate symptoms associated with stress [42,43]. A random-effects meta-analysis of controlled clinical trials evaluating the effects of prebiotics and probiotics on depression and anxiety revealed that probiotics yielded small but significant effects for depression and anxiety [44]. A recent study investigated the effects of probiotics on brain activity induced by emotional stimuli using functional MRI and reported that changes in brain activity induced by anxiety-evoking stimuli before and after the intervention were examined by MRI and showed a decrease in activity in anxiety-related regions induced by anxiety-evoking stimuli compared to a control group [45]. Probiotics may have caused changes in the interaction between the gut and the brain by regulating the gut environment. Further research is required to determine the effects of improving dysbiosis and alleviating constipation via SCFAs, such as butyric acid, on depressive symptoms.

In this study, reflex-related QOL score was also associated with the GDS-15 score. A prospective, observational cohort study using standardized, validated questionnaires, including the Hospital Anxiety and Depression Scale (HADS) revealed that patients with reflux symptoms have a high prevalence of anxiety [46]. The cross-sectional study utilizing data from a cohort of 10,000 adults revealed that smoking, alcohol intake, inactivity, high intake of sweets and desserts, low intake of fiber, depression, visceral fat, and obesity are considered risk factors for GERD [47]. In a previous study, we reported that upper abdominal symptoms as well as constipation are associated with frailty [48]. Recent research has revealed that gut frailty may be a worsening factor for various diseases, a cause of chronic inflammation, and a precursor to frailty [49]. Improving constipation and maintaining gut health may affect overall body and brain health.

Our research has certain limitations. First, the study population included only outpatients aged ≥ 65 years who were treated at a single university hospital specializing in geriatric medicine. Second, other background variables, such as lifestyle, activity level, exercise routines, dietary patterns, occupation, education level, and marital status, were not analyzed. Therefore, our results may not be applicable to all elderly individuals. This study is a cross-sectional study, so the causal relationship between depressive symptoms and constipation is unclear. Furthermore, there may be potential bias in our results due to the selection of unhealthy participants. Future intervention studies assessing the relationship between constipation and depressive symptoms are warranted.

## 5. Conclusions

Depressive symptoms in the elderly may be associated with various abdominal symptoms, particularly constipation. Multiple regression analysis revealed significant associations of GDS-15 scores with BMI and MMSE, constipation-related QOL, reflex-related QOL, diarrhea-related QOL, and CSS scores.

## Figures and Tables

**Table 1:** Clinical characteristics of study participants (n = 984).

Characteristics	Value
**Patient profile**	
**Male/Female**	**427 (43.4)/557 (56.6)**
**Age (years)**	**78.1 ± 6.1**
**BMI (kg/m^2^)**	**22.9 ± 3.7**
**Brinkman index**	**360.8 ± 612.7**
**Alcohol intake**	**0.5 ± 0.8**
**Use of therapeutic agents**	
**Statins**	
**Yes/No**	**412 (41.9)/572 (58.1)**
**Acid secretion suppressants**	
**Yes/No**	**494 (50.2)/490 (49.8)**
**Laxatives**	
**Yes/No**	**218 (22.2)/766 (77.8)**
**Neuropsychological examination**	
**MMSE**	**27 (25, 29)**
**GDS-15**	**4 (2, 6)**
**Gastrointestinal-related QOL score**	
**Reflex**	**1 (0, 3)**
**Upper abdominal pain**	**0 (0, 1)**
**Fullness**	**0 (0, 3)**
**Constipation**	**1.5 (0, 4)**
**Diarrhea**	**1 (0, 3)**
**Severity of constipation**	
**CSS score**	**2 (1, 6)**

Data are presented as number (%) or the mean ± standard deviation or median (lower quartile, upper quartile). MMSE, Mini-Mental State Examination; GDS-15, Geriatric Depression Scale 15; CSS, constipation scoring system.

**Table 2:** Clinical characteristics of study participants by GDS-15 severity.

	Normal	Mild	Moderate-Severe
	(n = 603)	(n = 319)	(n = 62)
**Age (years)**	**77.9 ± 6.0**	**78.5 ± 6.2**	**78.0 ± 6.1**
**Male**	**269 (44.6)**	**128 (40.1)**	**30 (48.4)**
**Female**	**334 (55.4)**	**191 (59.9)**	**32 (51.6)**
**BMI**	**23.0 ± 3.7**	**22.6 ± 3.7**	**22.7 ± 4.1**
**CSS score**	**2 (0, 4)**	**3 (1, 7)**	**5 (3, 8)**
**Reflux**	**0 (0, 2)**	**1 (0, 3)**	**2 (0, 5)**
**Upper abdominal pain**	**0 (0, 1)**	**0 (0, 2)**	**1 (0, 4)**
**Fullness**	**0 (0, 2)**	**1 (0, 4)**	**2.5 (0, 5)**
**Constipation**	**1 (0, 3)**	**2 (1, 4)**	**4 (2, 6)**
**Diarrhea**	**0 (0, 3)**	**2 (0, 4)**	**3 (0, 6)**
**GDS-15**	**2 (1, 3)**	**6 (5, 7)**	**11.5 (10, 12)**

Data are presented as number (%) or the mean ± standard deviation or median (lower quartile, upper quartile). GDS-15, Geriatric Depression Scale 15; CSS, constipation scoring system.

**Table 3:** Correlations between Geriatric Depression Scale 15 score and various clinical parameters.

Clinical Parameters	r	*p*-Value
**Age**	**0.028**	**0.190**
**BMI**	**−0.056**	**0.041**
**Brinkman index**	**0.049**	**0.063**
**MMSE**	**−0.092**	**0.002**
**Reflux**	**0.253**	**<0.001**
**Upper abdominal pain**	**0.229**	**<0.001**
**Fullness**	**0.269**	**<0.001**
**Constipation**	**0.329**	**<0.001**
**Diarrhea**	**0.264**	**<0.001**
**CSS score**	**0.285**	**<0.001**

r, Pearson’s correlation coefficients; BMI, body mass index; CSS, constipation scoring system; MMSE, Mini-Mental State Examination; QOL, quality of life.

**Table 4:** Associations between Geriatric Depression Scale 15 score and other variables in the multiple regression analysis.

	B	SE	95% CI of B		β	t	VIF	*p*-Value
**BMI**	−0.056	0.024	−0.103	−0.009	−0.069	−2.336	1.017	0.020
**MMSE**	−0.073	0.029	−0.131	−0.015	−0.074	−2.479	1.035	0.013
**Constipation**	0.161	0.053	0.057	0.265	0.136	3.043	2.352	0.002
**Reflux**	0.162	0.043	0.078	0.246	0.126	3.772	1.313	<0.001
**Diarrhea**	0.111	0.040	0.031	0.190	0.095	2.740	1.415	0.006
**CSS score**	0.079	0.033	0.015	0.144	0.098	2.403	1.963	0.016

BMI, body mass index; MMSE, Mini-Mental State Examination; CSS, constipation scoring system; SE, standard error; CI, confidence interval; VIF, variance inflation factor.

## Data Availability

Data supporting the findings of this study are available upon request from the corresponding author.

## References

[1] Arai H., Ouchi Y., Toba K., Endo T., Shimokado K., Tsubota K., Matsuo S., Mori H., Yumura W., Yokode M. (2015). Japan as the front-runner of super-aged societies: Perspectives from medicine and medical care in Japan. Geriatr. Gerontol. Int..

[2] Choung R.S., Locke GR3rd Schleck C.D., Zinsmeister A.R., Talley N.J. (2007). Cumulative incidence of chronic constipation: A population-based study 1988–2003. Aliment. Pharmacol. Ther..

[3] Bongers M.E., Benninga M.A., Maurice-Stam H., Grootenhuis M.A. (2009). Health-related quality of life in young adults with symptoms of constipation continuing from childhood into adulthood. Health Qual. Life Outcomes.

[4] Sumida K., Molnar M.Z., Potukuchi P.K., Thomas F., Lu J.L., Yamagata K., Kalantar-Zadeh K., Kovesdy C.P. (2019). Constipation and risk of death and cardiovascular events. Atherosclerosis.

[5] Asaoka D., Takeda T., Inami Y., Abe D., Shimada Y., Matsumoto K., Ueyama H., Matsumoto K., Komori H., Akazawa Y. (2020). The Association between Frailty and Abdominal Symptoms: A Hospital-based Cross-sectional Study. Intern. Med..

[6] Asaoka D., Takeda T., Inami Y., Abe D., Shimada Y., Matsumoto K., Ueyama H., Matsumoto K., Komori H., Akazawa Y. (2021). As-sociation between the severity of constipation and sarcopenia in elderly adults: A single-center university hospital-based, cross-sectional study. Biomed. Rep..

[7] Rao S.S., Rattanakovit K., Patcharatrakul T. (2016). Diagnosis and management of chronic constipation in adults. Nat. Rev. Gastroenterol. Hepatol..

[8] Towers A.L., Burgio K.L., Locher J.L., Merkel I.S., Safaeian M., Wald A. (1994). Constipation in the elderly: Influence of dietary, psychological, and physiological factors. J. Am. Geriatr. Soc..

[9] Zhao Y., Yu Y.B. (2016). Intestinal microbiota and chronic constipation. Springerplus.

[10] Zhao K.X., Huang C.Q., Xiao Q., Gao Y., Liu Q.X., Wang Z.R., Li Y.H., Xie Y.Z. (2012). Age and risk for depression among the elderly: A me-ta-analysis of the published literature. CNS Spectr..

[11] Riedel-Heller S.G., Luppa M. (2013). Depression in late life—What does epidemiology add?. Psychiatr. Prax..

[12] Morley J.E. (2003). Anorexia and weight loss in older persons. J. Gerontol. A Biol. Sci. Med. Sci..

[13] Zhang Y., Chen Y., Ma L. (2018). Depression and cardiovascular disease in elderly: Current understanding. J. Clin. Neurosci..

[14] Liu Y., Cui J., Cao L., Stubbendorff A., Zhang S. (2024). Association of depression with incident sarcopenia and modified effect from healthy lifestyle: The first longitudinal evidence from the CHARLS. J. Affect. Disord..

[15] Walther D.J., Peter J.U., Bashammakh S., Hörtnagl H., Voits M., Fink H., Bader M. (2003). Synthesis of serotonin by a second tryptophan hydroxylase isoform. Science.

[16] Agus A., Planchais J., Sokol H. (2018). Gut Microbiota Regulation of Tryptophan Metabolism in Health and Disease. Cell Host Microbe.

[17] Yano J.M., Yu K., Donaldson G.P., Shastri G.G., Ann P., Ma L., Nagler C.R., Ismagilov R.F., Mazmanian S.K., Hsiao E.Y. (2015). Indigenous bacteria from the gut microbiota regulate host serotonin biosynthesis. Cell.

[18] Matsuno K., Asaoka D., Sugano K., Takahashi K., Miyauchi K. (2024). Rationale and design of Juntendo Sarcopenia Registration to explore the predictors and prognosis of sarcopenia and frailty in the elderly in TOKYO (JUSTICE-TOKYO). Geriatr. Gerontol. Int..

[19] Harris P.A., Taylor R., Thielke R., Payne J., Gonzalez N., Conde J.G. (2009). Research electronic data capture (REDCap)—A metadata-driven methodology and workflow process for providing translational research informatics support. J. Biomed. Inform..

[20] Sugishita K., Sugishita M., Hemmi I., Asada T., Tanigawa T. (2017). A Validity and Reliability Study of the Japanese Version of the Ger-iatric Depression Scale 15 (GDS-15-J). Clin. Gerontol..

[21] Folstein M.F., Folstein S.E., McHugh P.R. (1975). “Mini-mental state”. A practical method for grading the cognitive state of patients for the clinician. J. Psychiatr. Res..

[22] Campbell K.E., Dennerstein L., Tacey M., Fujise N., Ikeda M., Szoeke C. (2017). A comparison of Geriatric Depression Scale scores in older Australian and Japanese women. Epidemiol. Psychiatr. Sci..

[23] Furuta K., Ishihara S., Sato S., Miyake T., Ishimura N., Koshino K., Tobita H., Moriyama I., Amano Y., Adachi K. (2009). Development and verification of the Izumo Scale, new questionnaire for quality of life assessment of patients with gastrointestinal symptoms. Nihon Shokakibyo Gakkai Zasshi.

[24] Agachan F., Chen T., Pfeifer J., Reissman P., Wexner S.D. (1996). A constipation scoring system to simplify evaluation and management of constipated patients. Dis. Colon Rectum.

[25] Dykes S., Smilgin-Humphreys S., Bass C. (2001). Chronic idiopathic constipation: A psychological enquiry. Eur. J. Gastroenterol. Hepatol..

[26] Nehra V., Bruce B.K., Rath-Harvey D.M., Pemberton J.H., Camilleri M. (2000). Psychological disorders in patients with evacuation disorders and constipation in a tertiary practice. Am. J. Gastroenterol..

[27] Cheng C., Hui W.M., Hu W.H., Wong N.Y., Lam K.F., Wong W.M., Lai K.C., Lam S.K., Wong B.C. (2005). Differing coping mechanisms, stress level and anorectal physiology in patients with functional constipation. World J. Gastroenterol..

[28] Emmanuel A.V., Mason H.J., Kamm M.A. (2001). Relationship between psychological state and level of activity of extrinsic gut innervation in patients with a functional gut disorder. Gut.

[29] He Z., Yu Q., He B., Liu J., Gao W., Chen X. (2024). Can depression lead to chronic constipation, or does chronic constipation worsen depression? NHANES 2005-2010 and bidirectional mendelian randomization analyses. BMC Gastroenterol..

[30] Vu N.T.H., Quach D.T., Miyauchi S., Luu M.N., Yoshida M., Nguyen D.T.N., Yoshino A., Miyaka Y., Okamoto Y., Oka S. (2024). Prevalence and associated factors of chronic constipation among Japanese university students. Front. Public Health.

[31] Bernstein A.L. (1990). Vitamin B6 in clinical neurology. Ann. N. Y. Acad. Sci..

[32] Hvas A.M., Juul S., Bech P., Nexø E. (2004). Vitamin B6 level is associated with symptoms of depression. Psychother. Psychosom..

[33] Tarracchini C., Lugli G.A., Mancabelli L., van Sinderen D., Turroni F., Ventura M., Milani C. (2024). Exploring the vitamin biosynthesis landscape of the human gut microbiota. mSystems.

[34] Kim S.E., Choi S.C., Park K.S., Park M.I., Shin J.E., Lee T.H., Jung K.W., Koo H.S., Myung S.J. (2015). Change of Fecal Flora and Effectiveness of the Short-term VSL#3 Probiotic Treatment in Patients With Functional Constipation. J. Neurogastroenterol. Motil..

[35] Ohkusa T., Koido S., Nishikawa Y., Sato N. (2019). Gut Microbiota and Chronic Constipation: A Review and Update. Front. Med..

[36] Takeda T., Asaoka D., Nojiri S., Yanagisawa N., Nishizaki Y., Osada T., Koido S., Nagahara A., Katsumata N., Odamaki T. (2023). Usefulness of Bifidobacterium longum BB536 in Elderly Individuals With Chronic Constipation: A Randomized Controlled Trial. Am. J. Gastroenterol..

[37] Macfarlane S., Macfarlane G.T. (2003). Regulation of short-chain fatty acid production. Proc. Nutr. Soc..

[38] Nakamori H., Iida K., Hashitani H. (2021). Mechanisms underlying the prokinetic effects of endogenous glucagon-like peptide-1 in the rat proximal colon. Am. J. Physiol. Gastrointest. Liver Physiol..

[39] Ge X., Zhao W., Ding C., Tian H., Xu L., Wang H., Ni L., Jiang J., Gong J., Zhu W. (2017). Potential role of fecal microbiota from patients with slow transit constipation in the regulation of gastrointestinal motility. Sci. Rep..

[40] Makizaki Y., Uemoto T., Yokota H., Yamamoto M., Tanaka Y., Ohno H. (2021). Improvement of loperamide-induced slow transit con-stipation by Bifidobacterium bifidum G9-1 is mediated by the correction of butyrate production and neurotransmitter profile due to improvement in dysbiosis. PLoS ONE.

[41] Sun J., Wang F., Hong G., Pang M., Xu H., Li H., Tian F., Fang R., Yao Y., Liu J. (2016). Antidepressant-like effects of sodium butyrate and its possible mechanisms of action in mice exposed to chronic unpredictable mild stress. Neurosci. Lett..

[42] Lalonde R., Violle N., Javelot H., Desor D., Nejdi A., Bisson J.F., Rougeot C., Pichelin M., Cazaubiel M., Cazaubiel J.M. (2011). Assessment of psychotropic-like properties of a probiotic formulation (Lactobacillus helveticus R0052 and Bifidobacterium longum R0175) in rats and human subjects. Br. J. Nutr..

[43] Kato-Kataoka A., Nishida K., Takada M., Kawai M., Kikuchi-Hayakawa H., Suda K., Ishikawa H., Gondo Y., Shimizu K., Matsuki T. (2016). Fermented Milk Containing Lactobacillus casei Strain Shirota Preserves the Diversity of the Gut Microbiota and Relieves Abdominal Dysfunction in Healthy Medical Students Exposed to Academic Stress. Appl. Environ. Microbiol..

[44] Liu R.T., Walsh R.F.L., Sheehan A.E. (2019). Prebiotics and probiotics for depression and anxiety: A systematic review and meta-analysis of controlled clinical trials. Neurosci. Biobehav. Rev..

[45] Tillisch K., Labus J., Kilpatrick L., Jiang Z., Stains J., Ebrat B., Guyonnet D., Legrain-Raspaud S., Trotin B., Naliboff B. (2013). Consumption of fermented milk product with probiotic modulates brain activity. Gastroenterology.

[46] Henning M., Lindgen K., Paul D., Fuchs C., Niecke A., Albus C., Bruns C., Pelzner K., Leers J. (2024). Association Between Anxiety and Reflux Symptoms in Patients With Gastroesophageal Reflux Disease: A Prospective Cohort Study. Cureus.

[47] Sadafi S., Azizi A., Pasdar Y., Shakiba E., Darbandi M. (2024). Risk factors for gastroesophageal reflux disease: A population-based study. BMC Gastroenterol..

[48] Asaoka D., Nomura O., Sugano K., Matsuno K., Inoshita H., Shibata N., Sugiyama H., Endo N., Iwase Y., Tajima Met a.l. (2024). Association Between Gastrointestinal-Related Quality of Life and Frailty Using Baseline Data of the Prospective Cohort Study (JUS-TICE-TOKYO Study). Diagnostics.

[49] Naito Y. (2024). Gut Frailty: Its Concept and Pathogenesis. Digestion.

